# Analysis of region specific gene expression patterns in the heart and systemic responses after experimental myocardial ischemia

**DOI:** 10.18632/oncotarget.17955

**Published:** 2017-05-17

**Authors:** Matthias Zimmermann, Lucian Beer, Robert Ullrich, Dominika Lukovic, Elisabeth Simader, Denise Traxler, Tanja Wagner, Lucas Nemec, Lukas Altenburger, Andreas Zuckermann, Mariann Gyöngyösi, Hendrik Jan Ankersmit, Michael Mildner

**Affiliations:** ^1^ Christian Doppler Laboratory for Cardiac and Thoracic Diagnosis and Regeneration, Medical University of Vienna, Vienna, Austria; ^2^ Department of Biomedical Imaging and Image-Guided Therapy, Medical University of Vienna, Vienna, Austria; ^3^ Department of Pathology, Medical University of Vienna, Vienna, Austria; ^4^ Department of Cardiology, Medical University of Vienna, Vienna, Austria; ^5^ Department of Thoracic Surgery, Medical University of Vienna, Vienna, Austria; ^6^ Department of Cardiac Surgery, Medical University of Vienna, Vienna, Austria; ^7^ Department of Dermatology, Research Division of Biology and Pathobiology of the Skin, Medical University of Vienna, Vienna, Austria

**Keywords:** myocardial infarction, systemic effect, paracrine factors, transcription factor, Klf4, Immunology and Microbiology Section, Immune response, Immunity

## Abstract

**Aims:**

Ischemic myocardial injury leads to the activation of inflammatory mechanisms and results in ventricular remodeling. Although great efforts have been made to unravel the molecular and cellular processes taking place in the ischemic myocardium, little is known about the effects on the surrounding tissue and other organs. The aim of this study was to determine region specific differences in the myocardium and in distant organs after experimental myocardial infarction by using a bioinformatics approach.

**Methods and Results:**

A porcine closed chest reperfused acute myocardial infarction model and mRNA microarrays have been used to evaluate gene expression changes. Myocardial infarction changed the expression of 8903 genes in myocardial-, 856 in hepatic- and 338 in splenic tissue. Identification of myocardial region specific differences as well as expression profiling of distant organs revealed clear gene-regulation patterns within the first 24 hours after ischemia. Transcription factor binding site analysis suggested a strong role for Kruppel like factor 4 (Klf4) in the regulation of gene expression following myocardial infarction, and was therefore investigated further by immunohistochemistry. Strong nuclear Klf4 expression with clear region specific differences was detectable in porcine and human heart samples after myocardial infarction.

**Conclusion:**

Apart from presenting a post myocardial infarction gene expression database and specific response pathways, the key message of this work is that myocardial ischemia does not end at the injured myocardium. The present results have enlarged the spectrum of organs affected, and suggest that a variety of organ systems are involved in the co-ordination of the organism´s response to myocardial infarction.

## INTRODUCTION

Myocardial infarction (MI) is defined as the death of cardiac myocytes due to prolonged ischemia. One of the most important interventions in the treatment of this cardiovascular disease is therefore protection of the affected ischemic area from apoptosis and necrosis [1, 2]. Early reperfusion strategies, accomplished through medical or mechanical means, are thus a mainstay of therapy and reduce mortality after acute MI by limiting infarct size [3, 4].

The recent evolution of high-throughput methods to investigate the transcriptome and proteome of cells and tissues provides new important insights into the mechanisms involved in the molecular processes induced after MI [5-16]. However, although great efforts have been made to unravel the molecular and cellular processes proceeding in the ischemic myocardium [17], little is known about the effects of MI on the surrounding tissue and other organs. Recently, Liu and co-workers used a rodent MI model to demonstrate that indeed myocardial ischemia can activate cardiac stem cells or mobilize progenitor cells. These cells then elicit global cardioprotective responses in distal organs, such as the bone marrow [18, 19] or liver [20], by regulating the expression of genes involved in the prevention of apoptosis and cell cycle progression [21]. It was further demonstrated that splenic monocytes are crucial for an adequate healing process after MI. They increase their motility, migrate from the spleen to the injured tissue and promote phagocytosis and wound healing [22-24]. In addition, splenectomy experiments indicate that this organ may contribute as much as half of the monocyte population recruited to the infarct zone [25]. Although there is increasing evidence that MI induces systemic processes that in turn act on the damaged area in the heart, little is known about the post-MI transcriptional gene expression profile in distal organs, such as the liver and spleen.

In the present study we used an experimental porcine MI model. Due to the high comparability to the human heart, the porcine closed chest reperfused acute MI model has been considered as one of the most relevant for studying molecular processes involved in MI [26-30]. The comparable physiology (relative heart size, cardiac and vascular anatomy and electrophysiology) [31, 32] and the fact that the experimental settings are more relevant to clinical conditions (no trauma due to thoracotomy and sternotomy) as compared to rodent models, has made the porcine model a valuable tool for myocardial research [33]. In addition, modern clinical settings, including coronary angiography, the investigation of revascularization and the MI-associated reperfusion-damage can be easily performed using this model [28].

By using the porcine closed chest reperfused acute MI model we aimed to (i) determine region specific differences in the myocardium after experimental MI (infarct zone, border zone and non-infarcted zone), (ii) describe molecular alterations caused by ischemic heart disease in distant organs (liver and spleen) and (iii) use up-to-date bioinformatics analyses to identify genes and molecular pathways that might play a central role in the systemic response to MI.

## RESULTS

### MI induces region-specific gene expression changes in the myocardium

**Figure 1:**
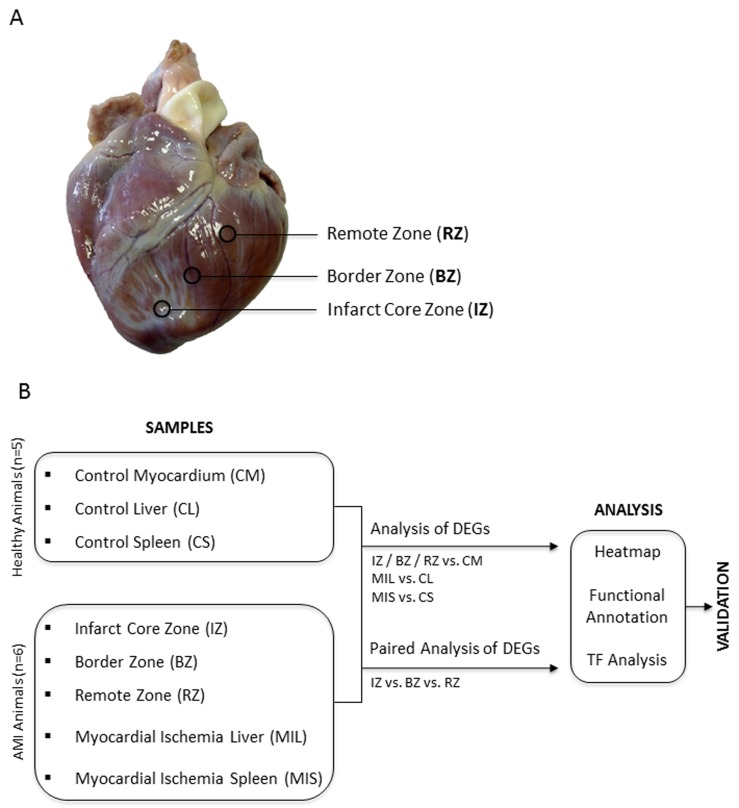
**A.** Biopsy Donor Site. Overview photography of a porcine heart after LAD occlusion shows the extension of affected myocardium and exact locations of three different myocardial regions respectively of the biopsy donor sites. **B.** Study design. DEG = differentially expressed genes; TF = transcription factor.

**Figure 2:**
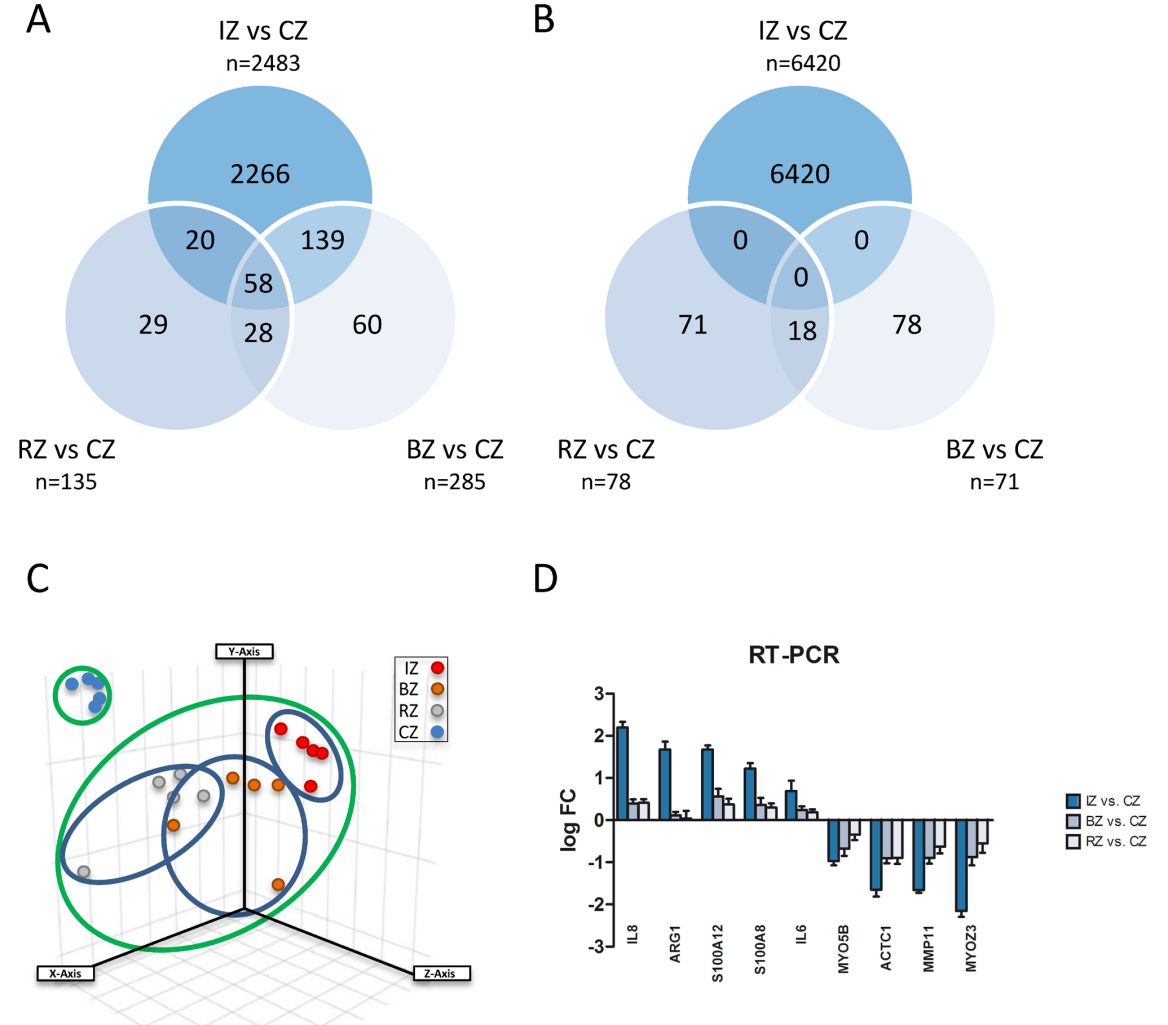
**A.** Venn diagram showing up-regulated genes with significant changes in all three areas of myocardium 24h after induction of acute myocardial infarction compared to control myocardium (CZ). Infarct core zone (IZ, *n* = 2483); border zone (BZ, *n* = 285); remote myocardium zone (RZ, *n* = 135). **B.** Venn diagram showing downregulated genes with significant changes in all three areas of myocardium 24h after induction of acute myocardial infarction compared to control myocardium (CZ). Infarct core zone (IZ, *n* = 6420); border zone (BZ, *n* = 71); remote myocardium zone (RZ, *n* = 78). **C.** Principal Component Analysis displaying mRNA expression data of the different areas of infarcted hearts and untreated healthy hearts. Infarct core zone (IZ, red dots); border zone (BZ, orange dots); remote myocardium (RZ, grey dots); control zone (CZ, blue dots). **D.** Validation of microarray results by RT-PCR. mRNA levels of nine selected genes were quantified by RT-PCR. Shown are mean ± SD of log FC values. RT-PCR data were normalized to beta-actin.

In order to evaluate gene regulation induced after experimental MI in the heart, we induced myocardial infarction in pigs and obtained samples of the core zone, border zone and remote myocardium (Figure 1) 24 hours after transient LAD occlusion. Subsequently, mRNA expression from the different areas of the infarcted heart was compared using the Agilent Whole Porcine Genome Oligo Microarray. As shown in Figure 2A+2B and additional files (Supplementary Data 2-4; adj. *P*-value < 0.005, fold change cut off < 2.0), we found 8903 differentially expressed genes in the core zone, 356 in the border zone and 213 in the remote zone as compared to control myocardium from healthy animals. Whereas in the border zone (135 up- *vs*. 78 down-regulated) and remote zone (285 up- *vs*. 71 down-regulated) more genes were up-regulated, the majority of genes in the infarct core zone (2483 up- *vs*. 6420 down-regulated) was down-regulated (Figure 2A, 2B). We displayed the mRNA expression data of the different areas of infarcted hearts and untreated healthy hearts in a principal component analysis (PCA; Figure 2C), and were able to cluster and separate all samples from each other according to the area of the heart. The greatest differences were observed between healthy hearts and all areas of the infarcted myocardium (Figure 2C). However, we could also clearly discriminate between all areas of the infarcted myocardium (Figure 2C, blue ovals), suggesting that MI also influences gene expression in the distant healthy parts of the heart. The highest quantitative changes in gene expression were observed in the infarct core zone. We further validated our microarray data by performing RT-PCR of selected genes strongly regulated in the chip analysis (Figure 2D), and found a high correlation between the two data sets (Supplementary Data 5).

### Functional annotation clustering of the regulated genes in the infarcted heart

**Figure 3:**
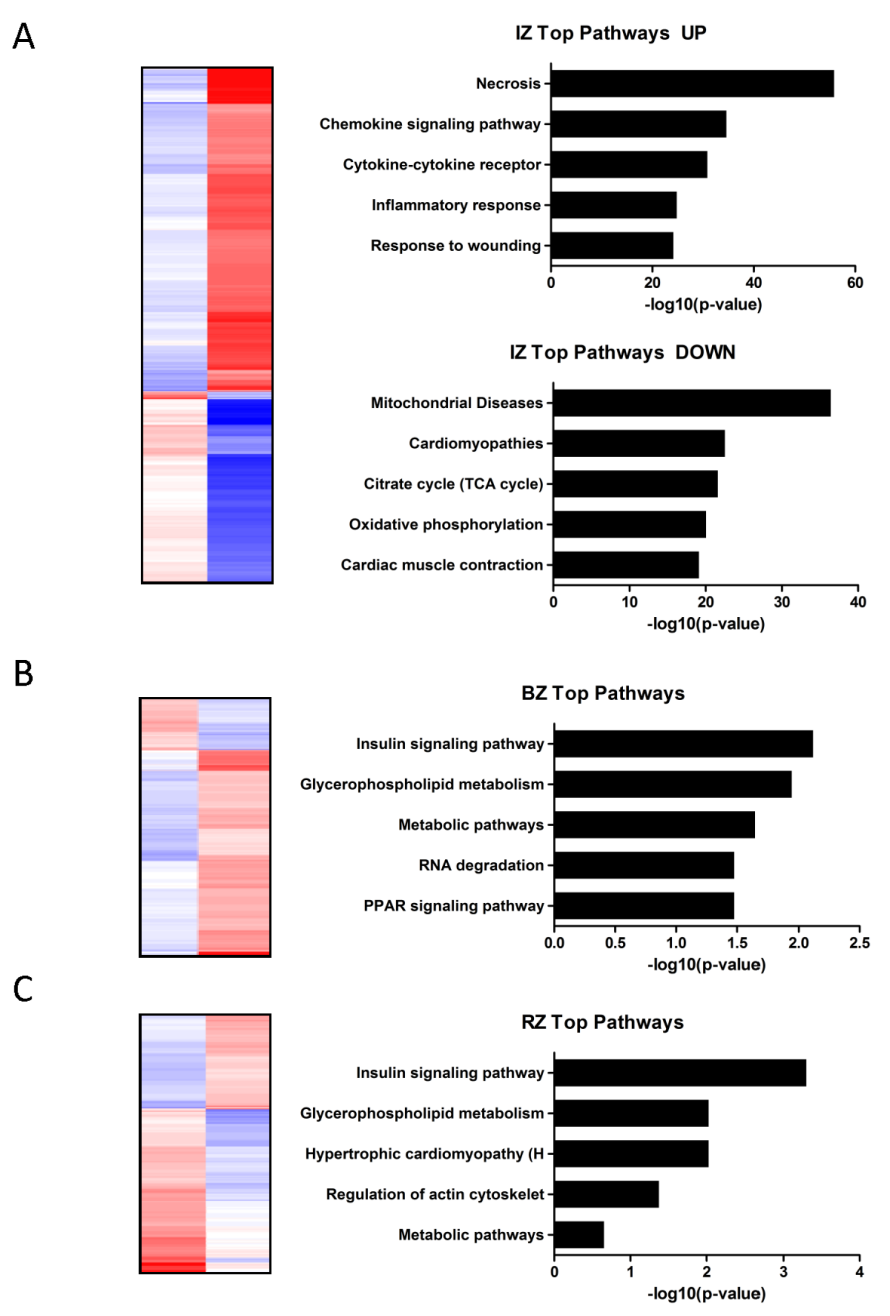
**A.-C:** Heat map showing the expression values (blue…upregulation; red…downregulation) of transcripts significantly altered (adjusted *P* value ≤ 0.05) in the infarct core zone **A**., border zone **B**. and remote zone **C**. compared to control myocardium. Functional annotation clustering of the regulated genes in the infarcted heart (A…up-/downregulated; B+C…all differentially regulated genes) identified pathways and biological processes affected by myocardial infarction. Pathways are sorted by decreasing *p*-values.

To identify pathways and biological processes affected in response to myocardial ischemia, functional enrichment analysis was performed. Assembling regional expression patterns yielded clusters that contained transcripts from multiple functional groups. According to the bioinformatics analysis genes up-regulated in the IZ were mainly associated with necrosis, chemokine signaling pathway, cytokine-cytokine receptor, inflammatory response and response to wounding (Figure 3A). These findings are in accordance with the concept that the inflammatory response is the first phase in infarct healing. The genes down-regulated in the IZ were associated with mitochondrial diseases, cardiomyopathies, citrate cycle, oxidative phosphorylation and cardiac muscle contraction (Figure 3A). In contrast to the IZ the molecular functions of significantly altered genes in the BZ (predominantly down-regulated genes) were essentially connected to insulin signaling pathway, glycerophospholipid metabolism, metabolic pathways, RNA degradation and PPAR signaling pathway (Figure 3B). In the RZ the main cardiovascular related pathways most commonly found were the insulin signaling pathway, glycerophospholipid metabolism, hyperthropic cardiomyopathy, regulation of actin cytoskelet and metabolic pathways (Figure 3C).

### Identification of over-represented transcription factor binding sites in sets of differentially expressed genes in myocardium post MI

**Figure 4:**
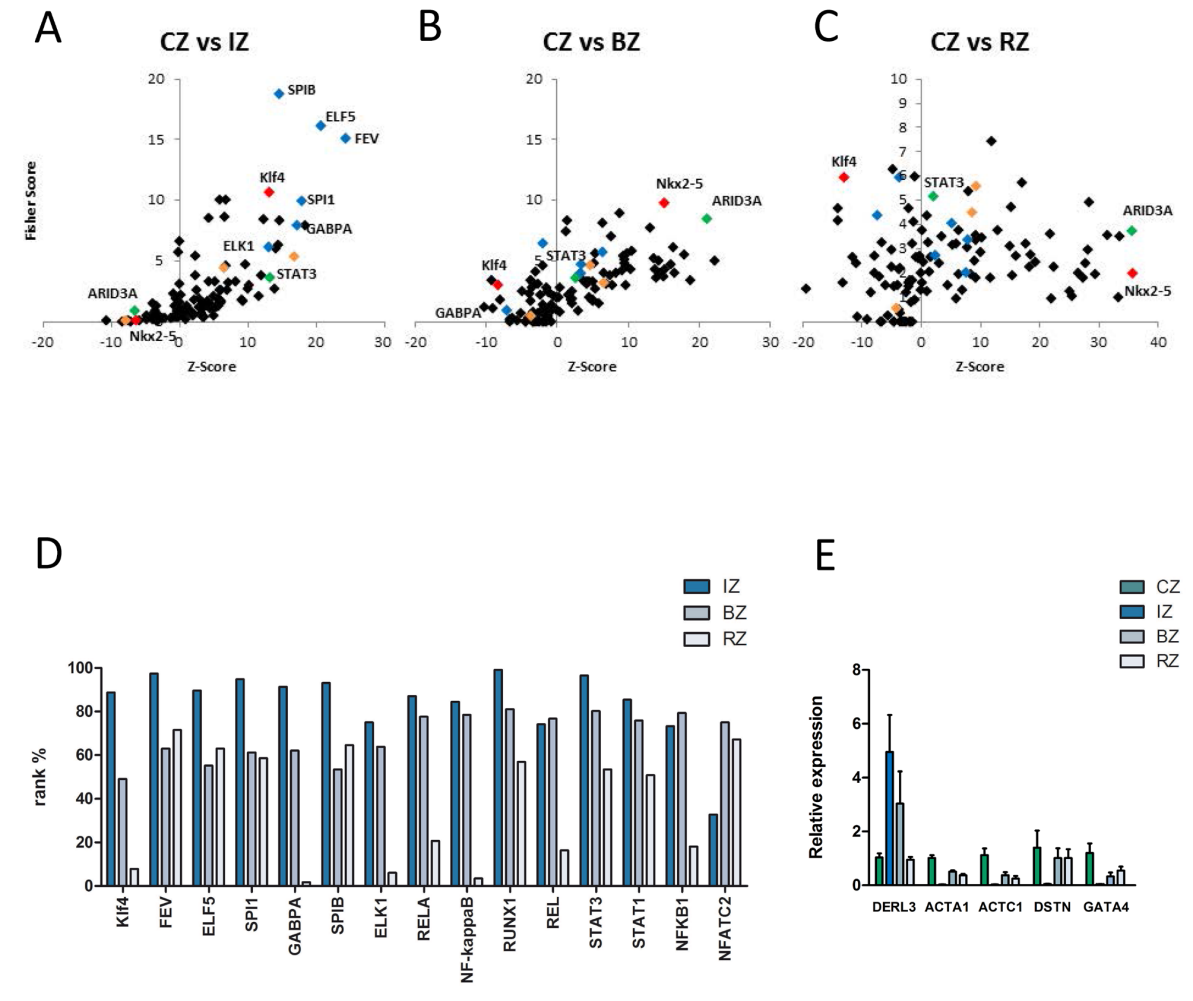
**A-C.** Identification of over-represented transcription factor binding sites (TFBS) in sets of differentially expressed genes in myocardium by oPOSSUM. **A.** In the infarct core zone members of the ETS transcription factor family (blue diamonds), key regulators of the hypertrophic transcriptional program (orange diamonds), signaling transducer and activator of transcription 3 (STAT3, green diamond) and kruppel-like factor 4 (Klf4, red diamond) binding sites were significantly enriched within the promoter regions. **B.** and **C.** In the border and remote zone AT-Rich Interaction Domain 3 (ARID3A, green diamond) and Nkx2-5 (red diamond) binding sites were significantly enriched within the promoter regions. **D**. Rank test of selected transcription factors based on the Fisher exact test score for different myocardial regions (IZ…infarct core zone, BZ … border zone, RZ … remote zone). A rank% value of 100 indicates that the TF mentioned are most probably functionally relevant. A rank% value of < 70 indicates that these TF may not be involved in gene regulation. Klf4 shows high values in the IZ and low values in the BZ and RZ. **E.** Five different down-stream targets of Klf4 show a significant up- (DERL3) and downregulation (ACTA1, ACTC1, DSTN, GATA4) of mRNA-levels in the infarct core-, border- and remote zone as compared to the control zone, measured by RT-PCR. Gene expression levels were calculated based on the mean value from six samples using the comparative Ct method and normalized to the housekeeping gene beta actin (ACTB). Data are presented as mean + SD.

oPOSSUM3 was used to identify over-represented transcription factor binding sites (TFBS) in the promoter sets of differentially expressed genes in all regions of the heart (Figure 4A-4C, Supplementary Data 6A-6C). Whereas the BZ and RZ showed a comparable enrichment of TFBS, the IZ displayed a completely different picture of overrepresented TFBS, suggesting a strongly altered transcriptional regulation of gene expression in the different myocardial regions.

**Figure 5:**
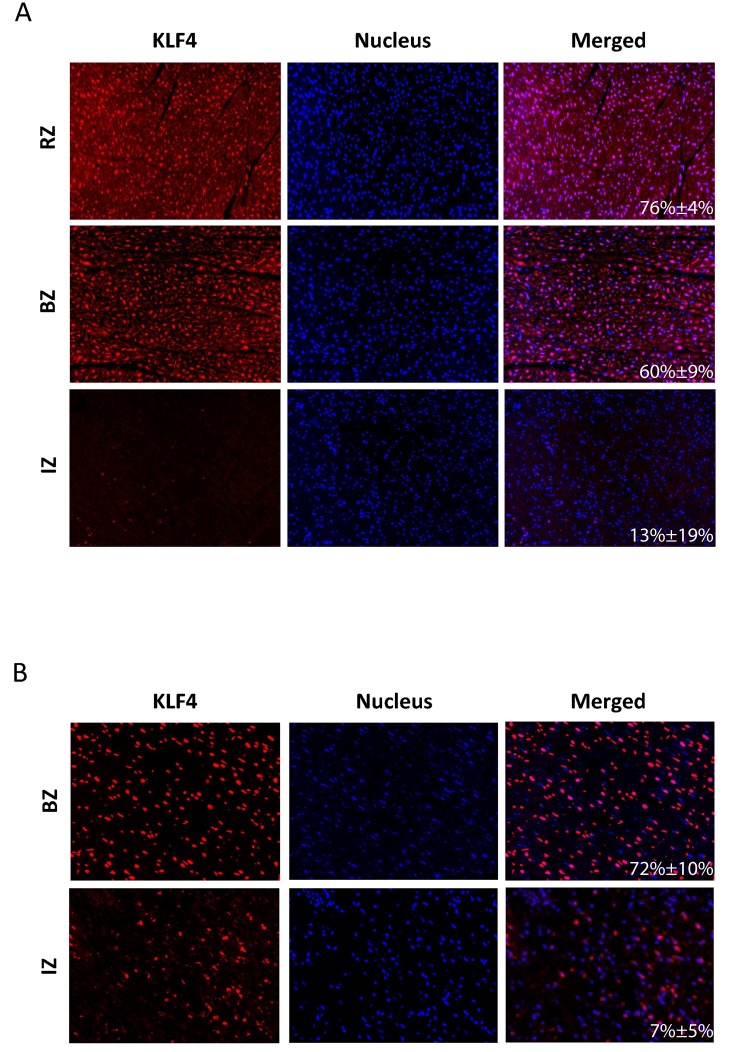
Representative IHC images of porcine **A.** and human **B.** left ventricles after myocardial infarction. Positive immunoreactive signals for Klf4 (red dots), nucleic acid (blue dots) and the merging of both stainings are shown for the infarct core zone (IZ), border zone (BZ) and remote zone (RZ). For human samples only tissue specimens from the site of infarction (IZ, BZ) were available. The mean percentage of Klf4 positive cells (±SD) is given in the merged picture.

Several members of the ETS transcription factor family (FEV, ELF5, SPIB, SPI1, ELK1, GABPA, blue diamonds), key regulators of the hypertrophic transcriptional program (NfkB, NFATs, MEF2, orange diamonds), signaling transducer and activator of transcription 3 (STAT3, green diamond) and kruppel-like factor 4 (Klf4, red diamond) binding sites were significantly enriched within the promoter regions of the infarct core zone (Figure 4A, 4D). In contrast, STAT3 and Klf4 could not be detected as major regulatory transcription factors in the BZ and RZ. In these regions AT-Rich Interaction Domain 3 (ARID3A, green diamonds) and Nkx2-5 (red diamonds) binding sites were significantly enriched within the promoter regions (Figure 4B-4D). The TF Klf4 displayed a linear gradient in our bioinformatics analysis, showing strongest activity in the IZ and weakest in the RZ (Figure 4D). We therefore asked whether we would be able to also reproduce these *in silico* data together with effects on Klf4 downstream targets in the *in vivo* situation. As shown in Figure 5A, nuclear Klf4 expression was readily detectable in the RZ in our porcine MI-model. By contrast, significantly fewer cells showed nuclear Klf4 expression in the BZ, and most of the cells of the IZ almost completely lost their Klf4 expression (Figure 5A). The mean percentage of Klf4 positive cells (±SD) was 76%±3.9% in the RZ, 60%±8.7% in the BZ and 13%±18.8% in the IZ. A significant difference exists between all three groups (*P*-value < 0.05). Similar regulation of nuclear Klf4 expression was observed in human heart samples after myocardial infarction (Figure 5B) with a mean percentage of Klf4 positive cells (±SD) of 72%±10.0% in the BZ and 7%±4.5% in the IZ (*P*-value < 0.001). We next examined whether the nuclear Klf4 expression regulated in the porcine hearts also affected Klf4 down-stream target genes. As shown in Figure 4E, five different down-stream targets of Klf4 showed a significant upregulation (DERL3) or downregulation (ACTA1, ACTC1, DSTN, GATA4) of mRNA-levels in IZ, BZ and RZ as compared to the CZ.

### MI induces systemic responses by regulating gene expression in the liver and spleen

**Figure 6A-6B:**
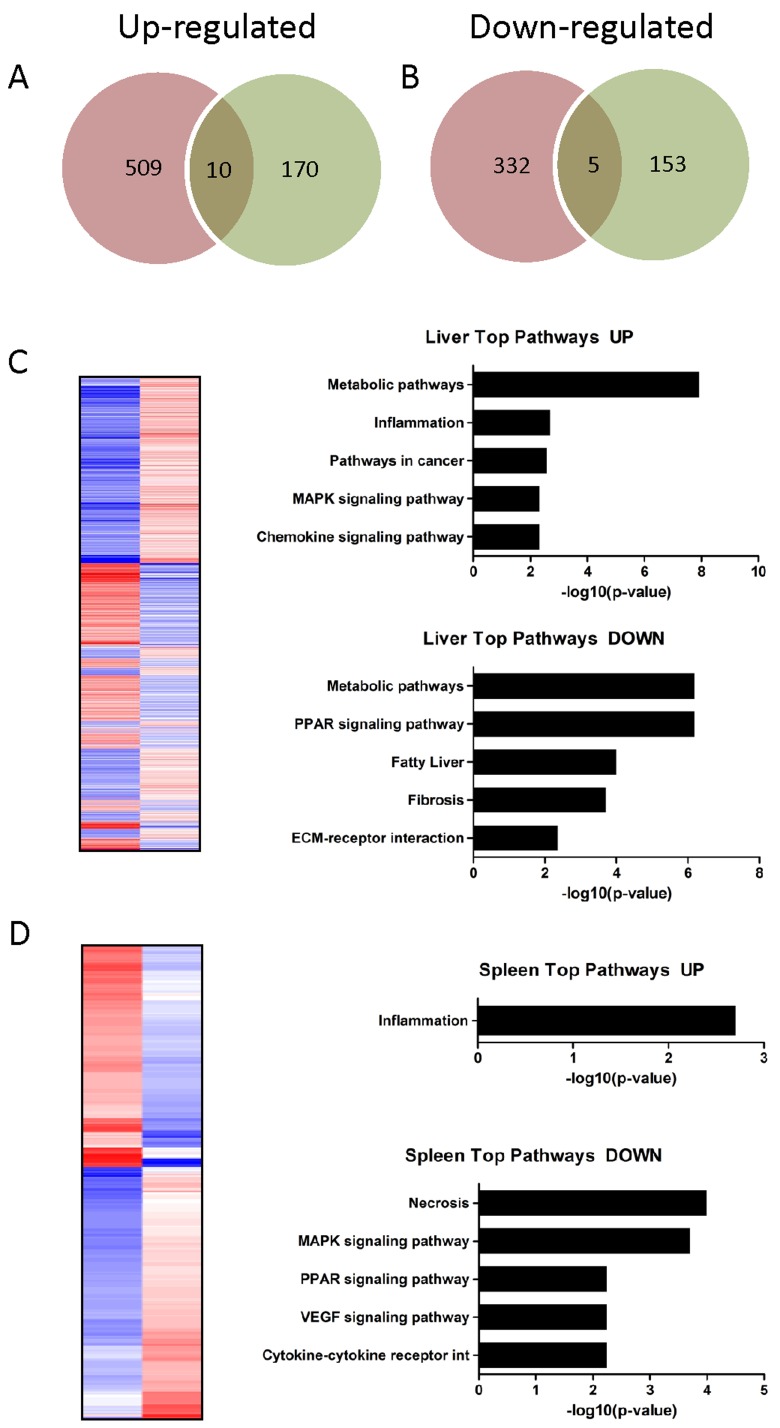
Venn diagram showing **A.** up- and **B.** downregulated genes in hepatic (red circle) and splenic (green circle) probe sets and the intersection of both tissue types. Among upregulated transcripts, ten genes could be identified, among downregulated transcripts five genes could be found in both probe sets. **C-D**: Heat map showing the expression values (blue = upregulation; red = downregulation) of transcripts significantly altered (adjusted *P* value ≤ 0.05) in hepatic **C.** and splenic **D.** probe **sets.** Functional annotation clustering of the up-/downregulated genes identified pathways and biological processes affected by myocardial infarction. Pathways are sorted by decreasing *p*-values.

To investigate whether MI induces a systemic response we investigated gene-regulation in the liver and spleen of infarcted animals. MI altered the expression of 856 genes in hepatic tissue (519 up- and 337 down-regulated genes) and 338 genes in splenic tissue (180 up- and 158 down-regulated genes) when compared to healthy animals. The Venn diagram in Figure 6 shows up- (A) and down-regulated (B) genes and the intersection of both tissue types. Among upregulated transcripts, 10 genes could be identified in both hepatic and splenic probe sets, among downregulated transcripts five genes could be found in both probe sets. The complete list of up- and down-regulated genes is given in Supplementary Data 7-8.

### Functional annotation clustering of the regulated genes in the liver and spleen

To identify pathways and biological processes affected in distal organs in response to MI we performed functional enrichment analysis from the microchip data of liver and spleen samples. The upregulated genes in the liver showed a strong association with metabolic pathways, inflammation, cancer associated pathways, and MAPK and chemokine signaling pathways (Figure 6C). 22 of these upregulated transcripts are annotated as “secreted” factors, indicating a secretory function of the liver post MI. The downregulated genes were associated with metabolic pathways, PPAR signaling pathways, biosynthesis of unsaturated fatty acids, fibrosis and ECM-receptor interaction. Again metabolic pathways were the predominant KEGG pathways (Figure 6C).

In spleen the majority of differentially expressed genes was downregulated and KEGG pathway analysis revealed a connection of these genes with necrosis, MAPK signaling, PPAR signaling and VEGF signaling pathways. (Figure 6D). The numerically smaller group of upregulated genes showed a strong association with inflammation (Figure 6D).

### Identification of over-represented transcription factor binding sites in sets of differentially expressed genes in hepatic and splenic tissue

**Figure 7:**
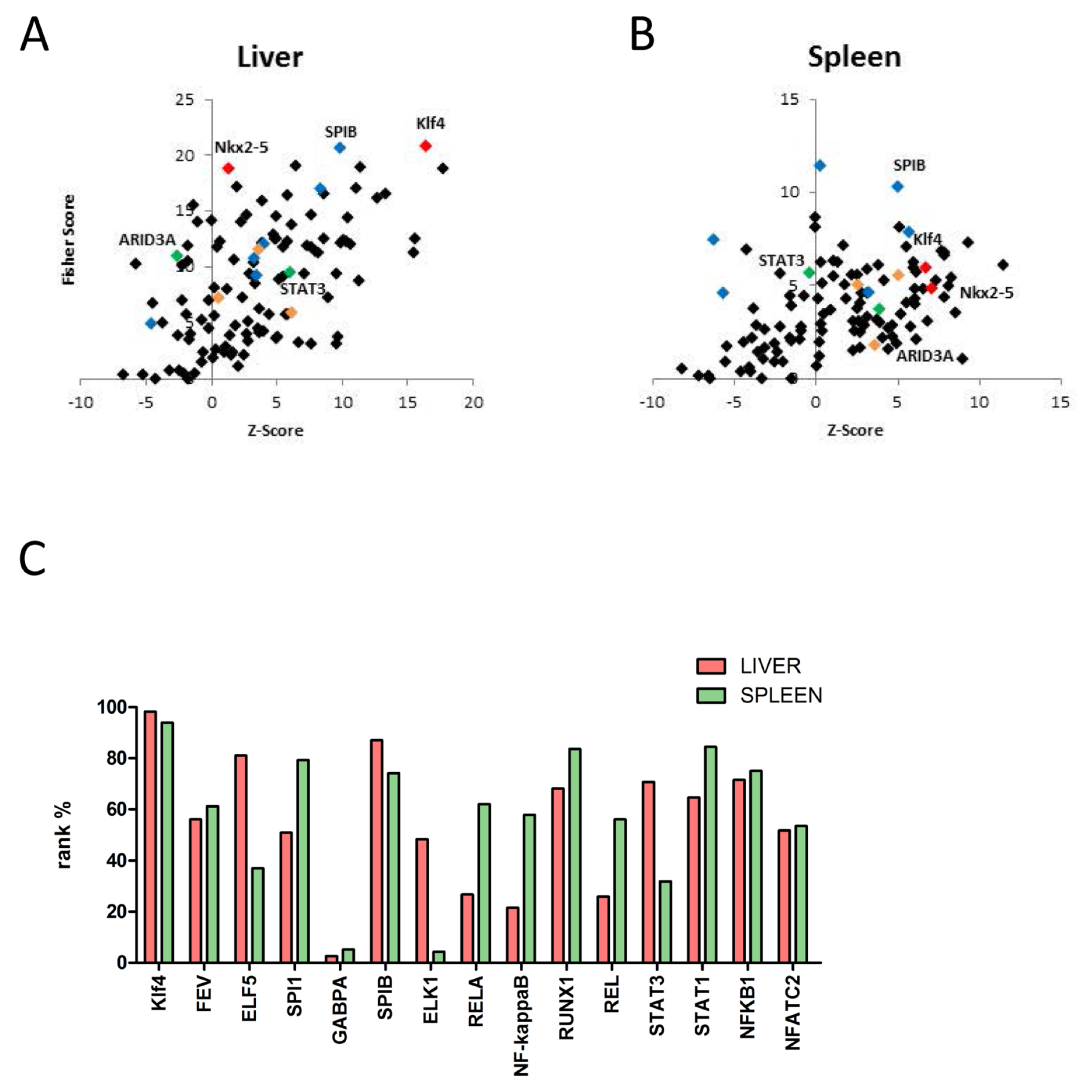
**A-B:** Identification of over-represented transcription factor binding sites (TFBS) in sets of differentially expressed genes in hepatic E and splenic F tissue by oPOSSUM. Members of the ETS transcription factor family (blue diamonds), key regulators of the hypertrophic transcriptional program (orange diamonds), signaling transducer and activator of transcription 3, (STAT3, green diamond), AT-Rich Interaction Domain 3 (ARID3A, green diamond), Nkx2-5 (red diamond) and kruppel-like factor 4 (Klf4, red diamond) binding sites are highlighted within the promoter regions. Binding sites for SPIB, Nkx2-5 and Klf4 are specifically enriched in the promoter regions of differentially regulated genes in both organs (liver and spleen) after MI. **C.** Rank test of selected transcription factors based on the fisher exact test score for liver and spenic tissue. A rank% value of 100 indicates that the mentioned TF are most probably functionally relevant. A rank% value of < 70 indicates that these TF may not be involved in gene regulation. Klf4 shows high values in both tissue types, comparable to those in the myocardial infarct core zone.

The TFBS analysis revealed that in both organs (liver and spleen) binding sites for SPIB, Nkx2-5 and Klf4 are specifically enriched in the promoter regions of differentially regulated genes after MI. Thus, a very similar picture of over-represented binding sites could be found in the liver and spleen, as well as in the remote zone of the heart, suggesting a general transcriptional regulation in many different cell types in ischemic heart disease (Figure 7A+7B; Supplementary Data 9A+9B)

## DISCUSSION

We have used mRNA microarrays to evaluate gene expression changes in the early period of myocardial ischemia in a porcine MI model. Identification of myocardial region specific differences as well as expression profiling of other distant organs revealed clear gene-regulation patterns within the first 24 hours after ischemia.

Cardiac hypertrophy as an adaptive response of the heart to hemodynamic and neurohumoral stress is one aspect of ventricular remodeling after AMI. This pathologic response occurs through the activation of molecular pathways. Over the past several years a number of nuclear factors have emerged as key regulators of the hypertrophic transcriptional program [34]. Here we provide evidence that MI induces a strong systemic response in distant organs and our bioinformatics approach identified several pathways and signaling molecules involved in the regulation of these processes.

### Region-specific gene expression changes in the myocardium

Ischemic myocardial injury results in decreased oxygen tension within the cell and subsequent loss of oxidative phosphorylation and decreased generation of ATP. Minutes after the onset of ischemia, reversible ultrastructural cardiomyocyte changes appear, including cellular and mitochondrial swelling and glycogen depletion. Cells dying by necrosis release their intracellular contents and initiate an intense inflammatory response by activating innate immune mechanisms [35]. Our pathway analyses of the most up- and down-regulated genes clearly identified changes in these cellular processes within the first phase after induction of MI (Supplementary Data 2-4). Downregulated genes reflected profound alterations in mitochondrial morphology, whereas upregulated genes played an important role in apoptosis and leukocyte recruitment. Transcriptional profiling revealed a finely adjusted expression balance between anti- and pro-inflammatory genes with neutrophils and monocytes/macrophages dominating the IZ. Interestingly, six out of the seven most differentially expressed IZ genes (IL8, ARG1, S100A12, CTSL, IL1R2, S100A8) identified in our study have been previously reported in myocardial ischemia [36-40], confirming the validity of our chip analysis.

Gene expression analyses of the border and remote myocardium demonstrated that these non-infarcted areas of the myocardium were also significantly affected and underwent remodelling after MI. Surprisingly, and in contrast to the IZ, most of the deregulated genes in the BZ and RZ were down-regulated (*n* = 58). Our bioinformatics analyses revealed a downregulation of the insulin signaling pathway and glycero-phospholipid metabolism in the BZ and RZ. Myocardial insulin resistance has been described two weeks after MI as a result of heart failure development [41]. However, using our analysis we identified changes in the myocardial insulin signaling pathway as early as 24 hours after induction of MI. Since insulin is known as an important regulator of cell cycle, cell survival and mitochondrial biogenesis [42], it is tempting to speculate that rapidly impaired insulin signaling in the BZ and RZ could contribute to an extension of the necrotic area after MI.

Multiple transcriptional regulators are required for proper cardiac function. Critical regulators include both cardiac-restricted transcription factors and co-regulators, most of which alter cell survival and differentiation [43]. Transcription factor binding site analysis of up- and down-regulated mRNAs in our experimental MI model identified a specific enrichment pattern of several putatively important transcription factors in the heart after MI. Many of these transcription factors have been shown to play crucial roles in pathophysiology of MI [44, 45] . Among them are the homeobox transcription factor Nkx2-5 and the zink finger transcription factors Klf4. Both Nkx2-5 and Klf4 work in a coordinated fashion with Gata4 to control a huge number of cardiac specific genes, including ANP, α-actin and ß-MHC [46-50]. In contrast to the essential role of Nkx2-5 and Klf4 during cardiac embryogenesis, its functional role in adult hearts has not been fully elucidated. Region specific analyses of the myocardium identified enrichment of TFBS for Klf4 in the IZ and Nkx2.5 in the BZ and RZ. We validated these data using qPCR to show that Klf4 is expressed in infarcted hearts and observed a strong downregulation of Klf4 in the IZ and BZ. Immunohistochemical analysis of Klf4 in porcine and human hearts was consistent with the PCR data, confirming our findings. Despite the fact that Klf4 has been implicated in apoptosis, data on Klf4 in ischemic disease are scarce. Our findings are contradictory to a recent publication by Zhang and coworkers [51] who showed an increased expression of Klf4 in injured cardiomyocytes following MI. However, in this study the authors did not distinguish between cytoplasmic and nuclear expression of Klf4 and the experiments were performed in mice. Our data clearly demonstrate that especially nuclear expression of Klf4 was strongly detectable in the non-infarcted areas of the heart (porcine and human) but not in the infarction zone. To further elucidate the role of Klf4 in ischemic disease we evaluated downstream targets of Klf4 by qPCR. We observed a strong downregulation of Acta1, Actc1 and Gata4 and Dstn and an upregulation of DERL3 specifically in the IZ. This is in line with previous publications showing that lack of Klf4 results in reduced expression of cardiac genes including Actc1 and Gata4 [52, 56]. In addition, DERL3 was implicated in the attenuation of stress response signaling and cell death in myocardial infarction [53].

Although several aspects of Klf4 in cardiomyocyte biology are not yet clearly understood, previous work provides evidence that Klf4 is a critical component in the transcriptional regulation of mitochondrial oxidative phosphorylation, biogenesis and the control of autophagy [54]. In the course of ischemia, mitochondria contribute to myocyte injury mainly *via* loss of their physiologic function, ultimately leading to contractile dysfunction of cardiomyocytes. Our finding that i) many of the downregulated genes in the infarcted area are associated with cellular processes such as mitochondrial homeostasis, citrate cycle or oxidative phosphorylation, and ii) putative target genes of Klf4 are downregulated, further emphasize the importance of this transcription factor for the homeostasis of the heart. In addition to mitochondrial function, Klf4 also regulates Gata4 gene expression in cardiac development and plays an essential role in regulating hypertrophic growth of the adult heart [55]. Furthermore, knock-down of Klf4 has been shown to compromise vascular integrity [56] and enhances hypertrophic phenotypes. In contrast, Klf4 over-expression significantly blocks cardiac hypertrophy [57]. These data, together with our findings suggest that Klf4 is a putative novel therapeutic target. Our data confirmed the importance of this transcription factor in the infarcted myocardium and identified a variety of other potentially important transcription factors which need to be further characterized.

### Systemic response after MI

Until now ischemic heart disease has been mainly considered as a cardiocentric process. Recent studies, however, suggested a more holistic view of this disease [58]. In addition to local inflammation, a profound systemic inflammation response has been documented in patients with AMI [59]. Following acute cardiomyocyte necrosis intracellular contents are released and an intense inflammatory response is initiated by activating innate immune mechanisms. Toll-like receptor (TLR)-mediated pathways, complement activation and reactive oxygen species generation play a significant role in triggering the postinfarction inflammatory response by activating the nuclear factor (NF-kB) system [35] .

**Figure 8:**
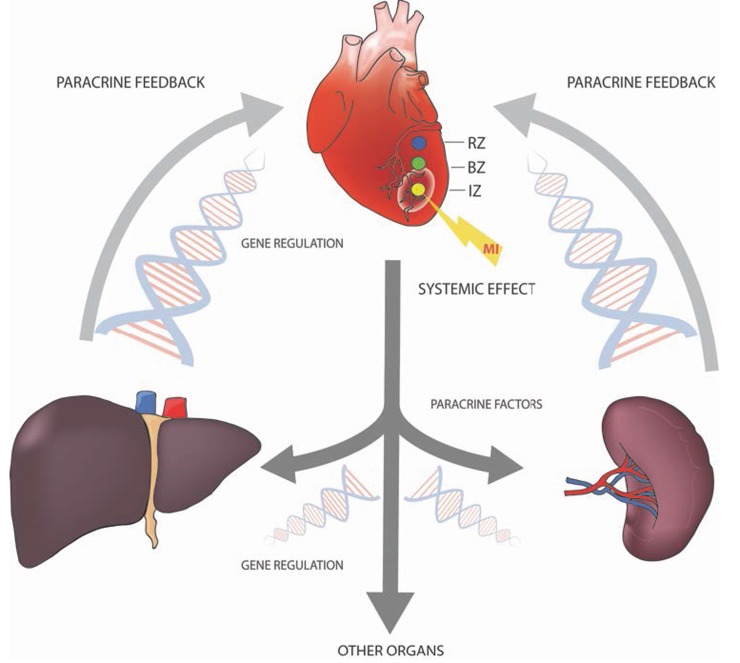
A variety of organ systems are involved in the coordination of the organism´s response to myocardial infarction by the production and release of paracrine factors For initiation of this paracrine feedback loops a variety of transcription factors are regulated in different organs.

Taking a genomic approach, we wanted to investigate how myocardial infarction elicits a global host response and whether there exists a crosstalk between the myocardium and spleen or liver (Figure 8). This inter-organ communication may be limited to leukocyte recruitment, but could comprise additional adaptive processes affecting injured myocardium. Analysis of the differentially regulated gene sets revealed that the majority of the 338 splenic and 856 liver transcripts were downregulated. This circumstance was reflected by reduced liver and splenic energy metabolism. PPAR signaling pathways, biosynthesis of unsaturated fatty acids, arachidonic acid metabolism, as well as MAPK signaling pathways were also significantly downregulated. These changes in energy metabolism may be due to changes in substrate concentrations or may be established to put the focus on the injured site. Furthermore we have found a clear disease association of differentially regulated genes, supporting the hypothesis of a systemic response to myocardial infarction. Liver tissue displayed an upregulation of genes involved in the regulation of cell death, inflammation and heart disease. 22 transcripts, encoding secreted proteins, were strongly upregulated in the liver after MI, suggesting that distal organs can be induced to release a plethora of paracrine factors, which in turn could confer cytoprotection to the damaged myocardium. These findings are in line with a recent publication by Liu and co-workers who demonstrated liver derived cardio-protective secretory proteins in a mouse model [60].

Transcription factor binding site (TFBS) analysis of up- and downregulated genes in hepatic and splenic tissue revealed two major points: i) the enrichment pattern was comparable in both tissues-types and ii) some of the identified TFs, including Klf4, seem to play a role in MI adaptational processes not only in the heart but also in other organs, such as liver and spleen. Our data emphasize that Klf4 is involved in cell differentiation, cell growth and cell cycle in multiple tissues. In the context of an inflammatory response after AMI and consistent with previous reports [61-63], Klf4 may be a critical regulator in the transcriptional network controlling lymphocyte differentiation.

## CONCLUSIONS

Transcriptional profiling is a useful approach to explore gene expression on a genome-wide scale, to create a global picture of specific cellular conditions and to generate testable hypotheses. The main goal of our study was the investigation of systemic responses of AMI in different organ systems, rather than the exact determination of AMI-induced effects on the single cell level. Although the animal model we used is closely related to human ischemic heart disease, more sophisticated experiments are needed to validate the pathways identified and their contribution to disease in the human system. Nevertheless we believe that the expression database presented here could contribute, together with other AMI models, to a greater understanding of the relationship between gene expression and cardiac function in normal and diseased hearts. Our data strongly suggest that myocardial ischemia does not end at the injured myocardium; instead, a variety of organ systems may be involved in the organism´s response to myocardial infarction.

## MATERIALS AND METHODS

### Porcine closed chest reperfused infarction model

A closed chest reperfused AMI infarction model was applied in a large animal setting after approval by the responsible local ethics committee (vote: 246/002/SOM2006). The experiments were carried out at the Institute of Diagnostics and Oncoradiology, University of Kaposvar, Hungary. We have used the AMI protocol from Nature protocols [64-66]. For the detailed protocol see Supplementary Materials and Methods (Supplementary Data 1).

### Tissue collection and RNA isolation

Isolated organs were washed with buffered saline to remove any residual blood. Samples of the myocardium were collected with a biopsy punch (6mm diameter) from three different areas: the core zone of infarcted area (IZ, middle of the scar of the left ventricle), the border zone (BZ, transmission between infarcted and surrounding tissue, perfused but hypocontractile) and the remote zone (RZ, non-infarcted myocardium of the left ventricle, perfused and functioning normally). The exact location of sample collection is shown in Figure 1. For details on RNA isolation see Supplementary Materials and Methods (Supplementary Data 1).

### Microarray gene expression analysis

Gene expression profiling services were performed by Miltenyi Biotec (Miltenyi, Bergisch-Gladbach, Germany). For the linear T7-based amplification step, 100ng of each sample was used. To produce Cy3-labeled cRNA, the RNA samples were amplified and labelled using the Agilent Whole Porcine Genome Oligo Microarray (one-color).

### Data analysis of microarrays

In order to analyse changes in gene expression caused by myocardial ischemia in the different regions, gene expression profiles of the infarct core zone (IZ), the border zone (BZ) and the remote zone (RZ) were compared with expression profiles of control myocardium from healthy animals.

Principal Component Analysis implemented in the GeneSpring software was used for the visual identification of data patterns and highlights similarities and differences between samples.

### Validation of microarray data by RT-PCR of selected genes

A selected set of nine genes, that were highly up-/ down-regulated in infarcted myocardium were validated with RT-PCR. For the detailed protocol see Supplementary Materials and Methods (Supplementary Data 1).

### Hierarchic clustering

GeneSpring software was used for hierarchic clustering of the miRNA expression data. An Euclidean distance metric and complete average-linkage clustering was used for hierarchic clustering.

### Statistical analysis of gene expression data

Details on statistical analysis are presented in the Supplementary Materials and Methods (Supplementary Data 1).

### Transcription factor binding site analysis

The web-based platform oPOSSUM3.0 (http://opossum.cisreg.ca/oPOSSUM3) was used for transcription factor binding site analysis [67]. For details see supplementary materials and methods (Supplementary Data 1).

### Functional annotation clustering and pathway analysis

Functional annotation clustering and pathway analysis was carried out as described previously [68]. For details see Supplementary Materials and Methods (Supplementary Data 1).

### Immunohistochemical staining in porcine and human hearts

Human heart tissue specimens (*n* = 5) have been collected during autopsy from subjects dying after acute myocardial infarction (AMI). Informed consent was obtained from family members as approved by the ethics committee of the Medical University of Vienna (vote: 2065/2016). For detailed staining procedure see Supplementary Materials and Methods (Supplementary Data 1).

## SUPPLEMENTARY MATERIALS
















