# Supplementary material for: Analysis of region specific gene expression patterns in the heart and systemic responses after experimental myocardial ischemia
Source: Oncotarget. 2017 May 17;8(37):60809–25. doi: 10.18632/oncotarget.17955 (PMC5617387; doi:10.18632/oncotarget.17955)
Supplement: Supplementary file 1 [file oncotarget-08-60809-s001.pdf]

Analysis of region specific gene expression patterns in the heart and systemic responses after experimental myocardial ischemia

SUPPLEMENTARY MATERIALS

Supplementary Data 1

Porcine closed chest reperfused infarction model

After overnight fasting, eleven domestic female pigs (weighing approximately 30 kg) were sedated with 12 mg/kg ketamine hydrochloride, 1.0 mg/kg xylazine and 0.04 mg/kg atropine. After the administration of 200 IU/kg of heparin, a 6F guiding catheter (Medtronic Inc., Minneapolis, USA) was introduced into the left coronary ostium, and selective angiography of the left coronary arteries was performed using Ultravist contrast medium (Bayer Healthcare, Berlin, Germany). A Maverick balloon catheter (diameter: 3.0 mm, length: 15 mm; Boston Scientific, Natick, USA) was inserted into the left anterior descending artery (LAD) after the origin of the second major diagonal branch. The LAD was then occluded by inflating the balloon slowly at 4–6 atm (n=6), controlling the occlusion with angiography. After 90 min of occlusion, the balloon was deflated and reperfusion was established. Control coronary angiography was performed to prove the patency of the infarct-related artery and to exclude arterial injury. Furthermore, all animals received 75 mg clopidogrel and 100 mg acetylsalicylic acid. After 24h euthanasia was performed by the administration of saturated potassium chloride and hearts, livers and spleens were immediately excised. Explanted hearts were sectioned into four slices perpendicular to the heart base-apex axis. Identification of the three different myocardial areas and sampling localization was made macroscopically. The equally thin fibrous scar area was considered to be the infarct core zone. The equally thick myocardial area proximal to the LAD occlusion segment (after second diagonal branch) was considered to be the non-infarcted remote zone. The transition zone between these two areas was considered to be the border zone. These decisions have been made based on experiences with 2,3,5-triphenyltetrazolium chloride (TTC) staining conducted in previous studies (Lichtenauer et al, Basic Res Cardiol. 2011). Principal component analysis confirmed that these areas were sampled correctly and the technique is feasible. Additionally appropriate control tissue from healthy animals (n=5) were included as physiological condition.

Mortality in the used animal model is primarily attributable to a predisposition for refractory arrhythmogenesis. Ventricular fibrillations only occurred within the first 30 minutes after induction of myocardial infarction. Due to the relatively small number of animals included in this study, no animals died within the 24 hours.

Tissue collection and RNA isolation

Biopsies from heart, liver and spleen were immediately collected in RNA later stabilization solution (Life Technologies), kept at 4°C overnight and stored at -80°C until RNA isolation. After homogenization of biopsies using a tissue homogenizer (Precellys, Bertin Instruments, Rockville, USA) total RNA was isolated using a miRNeasy Mini Kit (Qiagen, Hilden, Germany). Purity and quality of isolated RNA were assessed by RNA 6000 Nano assay on a 2100 Bioanalyzer (Agilent, Santa Clara, CA, USA). For all samples the RNA Integrity Number (RIN) was above eight and therefore optimal for microarray experiments.

Validation of microarray data by RT-PCR of selected genes

mRNAs of myocardial probes of all 4 regions were reverse transcribed to cDNAs (Qiagen, Hilden, Germany) and expression were quantified by RT-PCR (Applied Biosystems 7500 Real-Time PCR System, Life Technologies, USA). Primers were designed using Primer3 software. The expression rates of the target genes were normalized to the housekeeping gene beta actin (ACTB). The relative gene expression level was calculated using the delta Ct method. The expression changes were calculated relative to expression levels in the myocardium of healthy animals.

Statistical analysis of gene expression data

Background-corrected fluorescence intensity values were imported into GeneSpring v.11, log2-transformed, and then normalized by quantile normalization. The mean values of identical replicate probes on each chip were calculated by GeneSpring.

Statistically significant genes were identified by un-paired T-test with false discovery rate (Benjamini-Hochberg test) of ≤ 0.05.

The Agilent whole porcine genome oligo microarray uses different probes for some transcripts. For statistical analysis these transcripts were treated as distinct transcripts, whereas functional analysis was executed with summarized values for different probes of the same gene.

Transcription factor binding site analysis

oPOSSUM 3.0 detects predicted transcription factor binding sites in the promoter sequences of co-expressed genes in order to evaluate whether a transcription factor binding site is enriched within the gene set. Upstream sequences (2000 bp) of upregulated genes were analyzed using the default parameters in oPOSSUM 3.0 Sequence-based Single Site Analysis (SSA).

Functional annotation clustering and pathway analysis.

The differentially regulated transcripts were classified according to two web-based Gene Toolkits WEBGESTALT (http://bioinfo. vanderbilt.edu/webgestalt/analysis.php) and DAVID Bioinformatic Database (https://david.ncifcrf.gov/home.jsp). The toolkits employed gene ontology term enrichment (Go-term), the Kyoto Encyclopedia of Genes and Genomes (KEGG) Analysis and Disease Association Analysis to determine functional pathways. Up- and downregulated transcripts have been analyzed separately for the IZ, liver and spleen. For the BZ and RZ a separate analysis of up- and down regulated genes has not been appropriate due to a small number of up-regulated genes and therefore the analysis has been performed with all differentially regulated genes in each organ.

Immunohistochemical staining in porcine and human hearts

Human heart tissue specimens (n=5) have been collected during autopsy from subjects dying after acute myocardial infarction (AMI). Informed consent was obtained from family members as approved by the ethics committee of the Medical University of Vienna (vote: 2065/2016).

Klf4 staining of paraffin embedded tissues was performed after deparaffination with xylene and antigen retrieval by boiling in a microwave for 5 minutes in citrate-buffer (pH=6, Dakocytomation, Glostrup, Denmark). Non-specific staining was blocked by incubation with 10% normal goat serum for 1 h. The slides were incubated for 1 h at room temperature with a Klf4 antibody (1µg/ml Abcam, Cambridge, UK), diluted in PBS containing 2% bovine serum albumin (BSA) and 10% goat serum. To visualize Klf4, sections were incubated with a rabbit Fluor546 antibody (1:500; Alexa, Eugene, OR, USA) for 1 hour at room temperature. Counterstaining was performed with Hoechst and the slides were mounted with Fluoprep (bioMerieux, Marcy l'Etoile, France).

To quantify the Klf4 immunofluorescence stainings of the different human and porcine heart areas we counted Klf4 positive and all nucleated cells and calculated the percent of Klf4 positive cells. Statistically significant differences were identified by ANOVA and un-paired T-test.

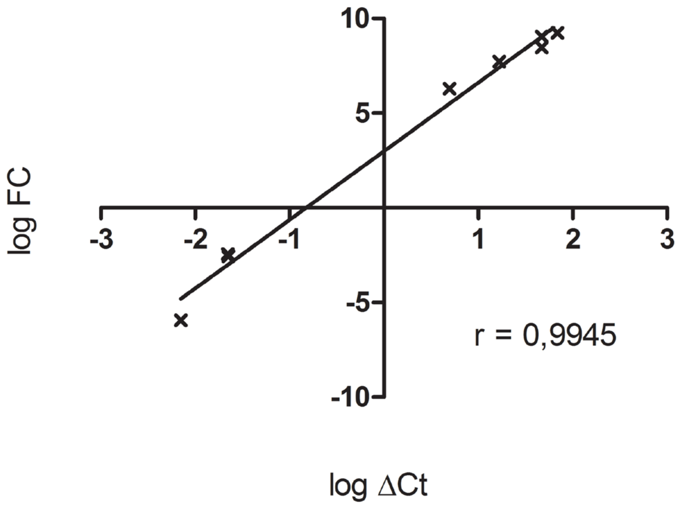

Supplementary Data 5: Correlation of RT-PCR and micrparray.

For Supplementary Data 2, 3, 4, 6, 7, 8, 9 see in Supplementary Files.
